# A Retrospective Analysis of the Challenges of Urothelial Cancer Management during the COVID-19 Pandemic at a Single Academic Center in Romania

**DOI:** 10.3390/healthcare11060812

**Published:** 2023-03-09

**Authors:** Vlad Barbos, Bogdan Feciche, Felix Bratosin, Iulia Bogdan, Rodica Anamaria Negrean, Silviu Latcu, Alexei Croitor, Vlad Dema, Razvan Bardan, Alin Adrian Cumpanas

**Affiliations:** 1 Department XV, Discipline of Urology, “Victor Babes” University of Medicine and Pharmacy, 300041 Timisoara, Romania; 2 Doctoral School, “Victor Babes” University of Medicine and Pharmacy, 300041 Timisoara, Romania; 3 Department of Urology, Satu-Mare County Emergency Hospital, 440192 Satu-Mare, Romania; 4 Department XIII, Discipline of Infectious Diseases, “Victor Babes” University of Medicine and Pharmacy, 300041 Timisoara, Romania; 5 Faculty of Medicine and Pharmacy, University of Oradea, 410073 Oradea, Romania; 6 Biochemistry Research Center, “Victor Babes” University of Medicine and Pharmacy, 300041 Timisoara, Romania

**Keywords:** COVID-19, urothelial cancer, cancer management, cancer epidemiology

## Abstract

The COVID-19 pandemic caused major changes in the healthcare sector due to adaptations required to hospitalize and treat an impressive number of patients. This retrospective study intended to collect reliable information on urothelial cancer patients in Romania. The primary objective was to compare the pre-pandemic and pandemic periods to observe the differences that occurred in the management of patients with urothelial carcinoma. The secondary objective was to determine the risk factors for urothelial cancer progression in the study cohort correlated with the COVID-19 pandemic. All patients that were diagnosed and treated at our clinic with a diagnosis of urothelial carcinoma (transitional cell carcinoma) during 2019–2021 were included in the current study. A total of 1122 eligible unique cases were identified during the study period. The number of patients who underwent intervention in the pre-pandemic year was 421, followed by a 22.6% decrease in 2020 to 326 cases and a 13.1% increase in 2021 to 375 cases. The proportion of muscle-invasive bladder cancer (MIBC) cases was significantly higher during the pandemic years, from 30.5% MIBC cases in 2019 to 37.4% in 2020 and 39.4% in 2021, suggesting a delay in presentations during the pandemic. Stage III and IV (TNM) cases were significantly more frequent, even though approximately 40% of all patients were operated on in stage I. The number of cystectomies increased significantly, from 5.2% in 2019 and 4.3% in 2020 to 10.1% in 2021, while the number of elective surgeries decreased, although no significant difference was observed regarding the in-hospital mortality and disease progression at six months. Patients with stage III and IV at presentation had the highest likelihood of disease progression at six months (HR = 5.61). Distant invasion was the second highest risk factor (HR = 5.13), followed by MIBC type (HR = 2.49). Nevertheless, the duration of hospitalization and year of diagnosis during the COVID-19 pandemic were not significant risk factors for cancer progression at six months. It can be concluded that there was a significant delay in patient presentations in 2020, and we advocate for increased public health awareness for urothelial cancer and increased attention toward the screening and management of these patients in the following years.

## 1. Introduction

The coronavirus disease 2019 (COVID-19) pandemic, triggered by the severe acute respiratory syndrome coronavirus (SARS-CoV-2) virus, has been lasting for almost three years, with a high morbidity and mortality burden [1]. In addition to the well-known pulmonary and systemic symptoms attributable to the virus, the high fatality numbers associated with SARS-CoV-2 infection have necessitated strategies to restrict its propagation and decrease hospitalizations, severe complications, and mortality rates [2,3]. Among these efforts to stop the pandemic were the strict quarantines, as well as modified health programs and policies for pandemic outbreaks that triggered many healthcare changes. The most significant healthcare shift has been in the operational elements of medical institutions [4]. As a result, the majority of outpatient appointments and procedures have been postponed, frequently without any preplanned patient or pathology-related methods, while medical practitioners have been recruited from their regular specialized medical field to practice COVID-oriented medicine [5,6].

As all the other medical specialties were in one way or another affected by the ongoing situation, the COVID-19 pandemic has influenced the urology practice as well, and the insights learned throughout the course of the pandemic should promote the development of suitable patient and pathology-related recommendations for current and future pandemics [7,8]. Since the first months of the COVID-19 outbreak, professional organizations have exerted great commitment to adapt to the worldwide pandemic effort in order to safeguard both doctors and patients. Patients with malignancies should be regarded as a population group with an increased danger of severe SARS-CoV-2 infection because of the neoplasia itself and the specialized care that often necessitates immunosuppressive medications and frequent hospitalizations [9,10,11]. Therefore, after the COVID-19 vaccines have proved their safety and effectiveness, immunization of the population of patients should be a primary priority [12,13] in an attempt to offer a further barrier against COVID-19 infection and avoid morbidity or death [14,15].

The postponement of surgical operations owing to the COVID-19 pandemic has reignited the issue in urological oncology over the permissible delay for invasive and non-invasive urothelial cancer therapy that involves mostly the bladder. A study from Austria determined that lockdowns during COVID-19 led to significantly fewer endoscopic procedures in the first half of 2020. Primary bladder cancer patients in 2020 had a significant increase in high-grade tumors and advanced stages compared to 2019. However, recurrent bladder cancer patients undergoing risk-adapted surveillance had no adverse outcomes regarding tumor stage and grade during the pandemic [16]. In the Netherlands, urothelial cancer diagnoses decreased by 14% during the first COVID wave but increased again in the second half of 2020. The decline was more pronounced in patients aged 70 and over and those with non-muscle-invasive bladder cancer [17]. Delays are detrimental irrespective of age, gender, or pathologic stage [18,19]. Consequently, radical cystectomy (RC) should be regarded as a postponed emergency rather than a normal treatment. Neo-adjuvant chemotherapy, where appropriate, allows saving some time until more favorable circumstances for proper surgical intervention [20,21,22], despite the fact that rigorous lockdown measures preclude non-emergent surgical operations. In addition, the detection of atypical histology during diagnostic transurethral resection of bladder tumor (TURBT) should be a reason for considering RC even more promptly [23].

During the SARS-CoV-2 pandemic, this study intended to collect reliable information on urothelial cancer patients in Romania. The main goal of the study was to observe the differences that occurred during the COVID-19 pandemic regarding the management of patients with urothelial carcinoma admitted to the urology department, while the secondary objective was to determine the risk factors for urothelial cancer progression in the study cohort.

## 2. Materials and Methods

### 2.1. Research Design and Ethical Considerations

The current study was designed as a retrospective cohort, aiming to collect data from patients diagnosed and treated for urothelial cancer at the Department of Urology of the Timis County Emergency Clinical Hospital “Pius Brinzeu” in Timisoara, Romania, during the 2019 to 2021 period. The Local Commission of Ethics for Scientific Research from the Timis County Emergency Clinical Hospital “Pius Brinzeu” from Timisoara, Romania, operates under article 167 provisions of Law no. 95/2006, art. 28, chapter VIII of order 904/2006; with EU GCP Directives 2005/28/EC, International Conference of Harmonisation of Technical Requirements for Registration of Pharmaceuticals for Human Use (ICH); and with the Declaration of Helsinki—Recommendations Guiding Medical Doctors in Biomedical Research Involving Human Subjects. The current study was approved on 11 October 2022, with approval number 333.

### 2.2. Inclusion Criteria

The inclusion criteria comprised all cases of urothelial cancer identified in the database of the Timis County Emergency Clinical Hospital “Pius Brinzeu” in Timisoara, Romania, during the 2019–2021 period. The hospital involved functions as a tertiary clinic for urological disease, with 65 hospital beds, serving a population of more than 600,000 in Western Romania. The main diagnosis of urothelial cancer (transitional-cell carcinoma) was used as a keyword for hospital database searches based on the International Classification of Diseases (ICD-10) [24]. After identifying all adult patients (>18 years old) with urothelial cancer, their paper records were reviewed to confirm the diagnosis and collect all the other relevant medical information. The diagnosis of urothelial carcinoma was considered valid only after a histopathology result. Patients with SARS-CoV-2 infection during hospitalization or during oncological treatment were also included in the study, and the COVID-19 diagnosis was considered only when a positive PCR test was present. Patients were excluded from the current study if they did not align with the following criteria: (1) providing a signed consent in the paper records for their willingness to provide personal data for medical research; (2) if their oncological diagnosis identified in the database did not match the diagnosis in the paper; (3) if the patient was underage; (4) missing relevant data of study variables considered for this study; (5) being diagnosed with urothelial carcinoma before 2019. The entire cohort of patients was further separated into three main study groups to observe the epidemiology and evolution of urothelial cancer before the pandemic (2019), during the first year of the pandemic (2020, including January and February), and during the second year of the pandemic (2021).

### 2.3. Variables

Based on the existing database and patient’s paper records, the current study identified the following relevant study variables: (1) patients’ background—age, age range, gender, substance use behavior, place of origin, referral source, COVID-19 vaccination status, SARS-CoV-2 infection; (2) urothelial cancer characteristics—number of comorbidities, anatomical distribution, tumoral infiltration, histological type, grading, tumor node metastasis (TNM) staging, patient performance status, and tumoral invasion; (3) urothelial cancer management features—intervention type, emergency presentation, and disease outcomes.

### 2.4. Statistical Analysis

The statistical analysis was performed with IBM SPSS v.27 (SPSS. Inc., Chicago, IL, USA), while the significance threshold was set for an alpha value of 0.05. The absolute and relative frequencies of categorical variables were computed and compared using the Chi-square and Fisher’s tests. The comparison of mean rank differences among nonparametric variables was performed with the Kruskal–Wallis test. Parametric continuous variables that followed a normal distribution were compared by mean and standard deviation with the Student’s t-test and analysis of variance (ANOVA) test for more than two groups. The Cox regression analysis was used to identify the hazard ratio for disease progression. A Kaplan–Meier analysis curve was plotted to determine the disease-free survival at six months for the studied patients.

## 3. Results

**Figure 1:**
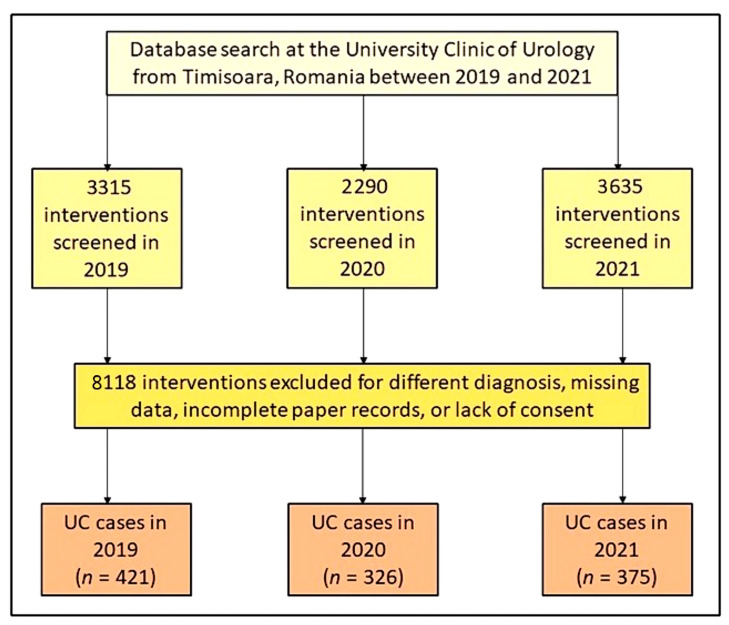
Diagram depicting the inclusion of patients who had surgery for urothelial carcinoma (UC) during the three-year data analysis.

The current study ended with the collection of data from a total of 1122 unique patients that underwent urological interventions during the three-year period of 2019, 2020, and 2021, as seen in Figure 1. It was observed that the number of interventions decreased dramatically in 2020 during the first year of the COVID-19 pandemic, from 3315 interventions in the clinic in 2019 to 2290 interventions in 2020, resulting in a 30.9% decrease. The following year 2021, the number of interventions increased by 37.0% to a total of 3635 interventions, which was even higher than the year preceding the pandemic. Therefore, it is hypothesized that patients who did not present in 2020 accumulated the following year in 2021. The number of patients with urothelial carcinoma (transitional cell carcinoma) in 2019 was 421, followed by a 22.6% decrease in 2020 to 326 cases and a 13.1% increase in 2021.

### 3.1. Patients’ Baseline Characteristics

The baseline characteristics of patients included in this study showed no significant difference between the age of patients who presented each year or the age range. The mean age was 63.2 years in 2019, 64.0 years in 2020, and 64.4 in 2021, with over 40% of patients in each group being in the 50–70 age range. Most of the patients were men, and the average body mass index was higher than 26.5, with no significant differences between the three studied years. It was observed that smoking had a high prevalence rate among patients with UC, with more than 35% of the entire cohort admitting to being a chronic smoker. Also, the majority of patients were living in the urban region (55.3%). Regarding the referral source, it was observed that a higher number of patients during the first year of the COVID-19 pandemic were referred from secondary care (61.0%), compared with 2019 and 2021, with 53.9% from secondary care. Also, there were 30 patients who were infected with SARS-CoV-2 during their hospital stay in the urology department. Lastly, a total of 11.3% of patients were vaccinated against COVID-19 by the end of 2020, increasing to 25.1% in 2021, as described in Table 1.

### 3.2. Oncological Features

**Figure 2:**
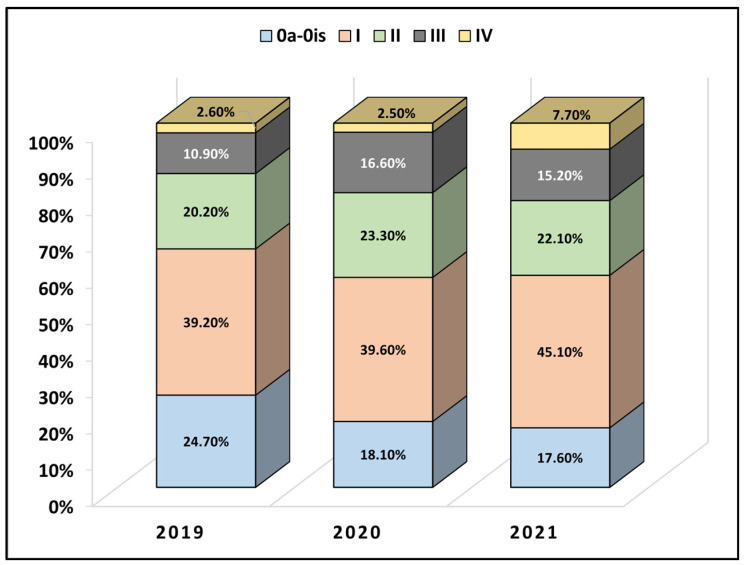
Comparative analysis of the TNM staging of urothelial carcinoma in patients receiving surgery before (2019) and during the SARS-CoV-2 pandemic (2020–2021).

Table 2 presents the medical history and oncological features of the studied patients with urothelial cancer. It was observed that approximately 80% of the entire cohort had bladder cancer, with the remaining 20% involving the renal pelvis and ureter, without any significant differences between the three studied years. However, regarding the proportion of cases with tumoral infiltration in the muscular layer, there was a significant increase during the pandemic years, from 30.5% muscle-invasive bladder cancer (MIBC) cases in 2019 to 37.4% in 2020 and 39.4% in 2021 (*p*-value = 0.046), suggesting a delay in presentations during the pandemic. Nevertheless, tumoral grading did not differ between these three years, with low tumor grading being the most prevalent. Comparable with the proportion of patients with MIBC, the TNM staging was also significantly different during the COVID-19 pandemic, when patients with stage III and stage IV transitional cell cancer were significantly more frequently observed (*p*-value < 0.001). There were 10.9% of patients with stage III cancer in 2019, compared with 16.6% in 2020 and 15.2% in 2021. Similarly, there were 7.7% of patients with stage IV cancer in 2021, compared with only 2.6% in 2019 and 2.5% in 2020. Stage I cancer patients were the most frequently observed (39.2% vs. 39.6% vs. 45.1%), as seen in Figure 2. Lastly, the local invasion was significantly more frequent among patients operated on during the second year of the pandemic (7.7% in 2021 vs. 2.5% in 2020 and 2.6% in 2019, *p*-value < 0.001).

### 3.3. Comparison of Outcomes and Interventions

It was observed that in 2021 the number of cystectomies increased significantly, from 5.2% in 2019 and 4.3% in 2020 to 10.1% in 2021 (*p*-value = 0.003). Consequently, the number of TURBT interventions decreased from 87.4% in 2019 to 77.9% in 2021, as seen in Table 3. Probably the reason behind this was the significantly increased amount of emergent presentations during the COVID-19 pandemic, instead of more electives as before 2020. There were 20.5% and respectively 19.4% emergency presentations, such as urinary obstruction and hemorrhagic cystitis due to urothelial cancer, compared to only 14.0% before the pandemic (*p*-value = 0.038). It was also observed that the duration of hospitalization decreased significantly during the COVID-pandemic, from an average of 9.8 days to 8.3 days in 2020 and 8.5 days in 2021 (*p*-value < 0.001). However, the in-hospital mortality rate and disease progression at six months were not statistically significant, even though they increased from 8.8% to 10.7% in 2020 and 11.7% in 2021. The mortality rate by type of intervention identified that 24 (55.8%) of patients who underwent nephrectomy died, compared to 13 (30.2%) after cystectomy and 6 (13.9%) after TURBT.

### 3.4. Risk Factor Analysis

**Figure 3:**
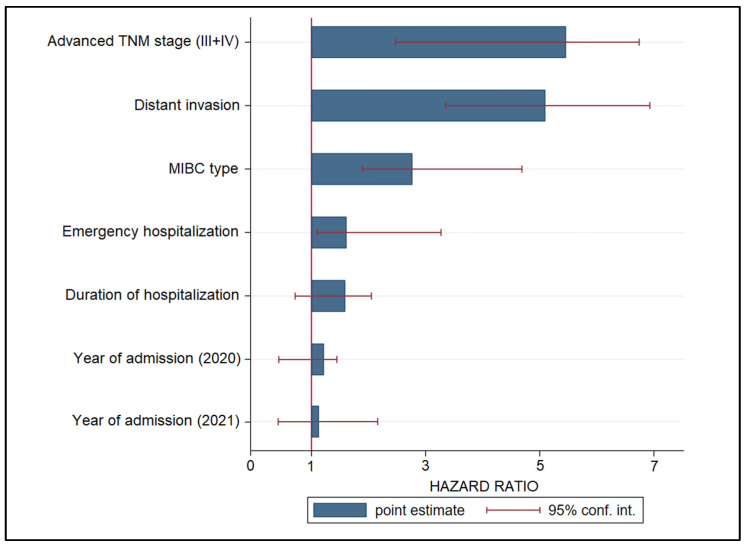
Analysis of risk factors for disease progression in patients with urothelial cancer.

The risk factor analysis for urothelial cancer progression at six months, presented in Table 4 and Figure 3, identified stages III and IV at presentation as having a higher likelihood of progressing, with a hazard ratio (HR) of 5.61 (*p*-value < 0.001). Distant invasion was the second highest risk factor (HR = 5.13), followed by MIBC type of cancer (HR = 2.49) and emergency hospitalization at diagnosis of UC (HR = 1.32). Nevertheless, the duration of hospitalization and year of diagnosis during the COVID-19 pandemic did not represent significantly high enough risk factors for cancer progression at six months.

## 4. Discussion

### 4.1. Important Findings and Literature Review

This study explored how the SARS-CoV-2 pandemic influenced the diagnosis and treatment of urothelial (transitional-cell) carcinoma in Romania. The current SARS-CoV-2 pandemic has resulted in a significant reduction in medical care for a substantial number of patients with cancer, corresponding with the experts’ projections and previous reports. Furthermore, a substantial proportion of patients initially diagnosed with a curable stage of urothelial cancer in its initial phases may have become untreatable in its later phases due to omitted consultations for urothelial cancer and elective surgery, intentional refusal of treatment, or intentional delay of treatment due to fear of SARS-CoV-2 virus.

In the context of patients recently diagnosed with urothelial cancer, early recommendations during the SARS-CoV-2 pandemic were motivated by the fear of contracting COVID-19 rather than a complete health evaluation assessing the morbidity and mortality attributed to urothelial neoplasia [25]. Comparing early cystectomy for high-risk urothelial carcinoma vs. delayed cystectomy [26] revealed that the effect of operative postponement was as significant in muscle-invasive bladder cancer (MIBC). Tumor histology seems to have a superior prognostic significance than operative treatment timeline in cases with high risk. Regarding low-grade NMIBC, solid evidence supports the safety of deferring cystoscopic monitoring and TURBT for patients with documented low-grade relapse until new symptoms develop. Regarding intravesical instillations in non-muscle-invasive bladder cancer NMIBC, there are insufficient data regarding the impact of delays, but repeated hospital visits predispose the patients to contract COVID-19 [27]. Consequently, these patients should get their induction dosages as soon as feasible, contrary to the early British recommendations [25] that recommended delaying Bacillus Calmette-Guérin (BCG) intravesical instillations out of concern for immunosuppression. Nevertheless, maintenance medication may be discontinued after three months if the risk of COVID-19 infection has decreased [28,29]. If patients who undergo intravesical BCG therapy are discovered to be infected with SARS-CoV-2, there is data promoting the interruption of instillations for three weeks to allow for full recovery, followed by treatment completion for six weeks, with a total duration of therapy not to exceed one year [30].

Furthermore, restrictive stay-at-home directives and the deferral of elective diagnostic procedures such as cystoscopy have delayed the identification of urothelial cancer. Delays surpassing 14 days between symptom development and diagnosis have a deleterious influence on survival since symptomatic bladder cancer is associated with a more advanced tumor stage [31]. Therefore, healthcare authorities should suggest virtual replacements when shutting down medical facilities (mainly telemedicine). These closures encourage individuals to manage their symptoms rather than seek medical attention. Virtual methods of consultation would enable the establishment of an effective approach for symptom evaluation while minimizing physical congestion in outpatient departments [32,33,34].

Concerning upper tract urothelial carcinoma, various studies have indicated that treatment rescheduling is associated with a poorer prognosis, including worse pathologic staging and degree of tumoral infiltration. The time of the intervention should be determined based on the oncological risk profile [35]. For instance, ureteral tumors have a poorer prognosis than renal pelvic tumors when surgery is postponed for more than one month. However, patients with pT2 or higher urothelial carcinoma did not have inferior survival results when their operation was postponed for more than three months, while similar findings were found for survival without metastasis and recurrence [36,37,38].

Similarly to our findings, it was observed in other studies that elective procedures for urothelial cancer conducted in the first year after the pandemic decreased significantly. Despite the fact that previously published data has revealed a progressive drop in the frequency of urothelial cancer during the past 20 years, the estimated fall of approximately 1% per year cannot account for the considerable reduction in urothelial cancer seen in other studies [39]. The protracted periods of lower elective operations seen in many hospitals after the COVID-19 outbreak may account for this outcome. Nevertheless, wait times for those undergoing urothelial cancer surgery did not rise considerably during the two time periods, indicating that fewer people have been showing urothelial cancer since the COVID-19 outbreak. However, in a recent systematic review, it was concluded that a prolonged interval between bladder cancer diagnosis and RC was significantly associated with poorer overall survival outcomes, emphasizing the importance of timely surgical intervention in the management of urothelial cancers [40]. Nonetheless, this effect was not evident in patients who received neoadjuvant chemotherapy prior to RC, implying that such treatment might help counteract the negative impact of delayed surgery. Furthermore, the study revealed that delaying radical nephroureterectomy following upper tract urothelial carcinoma diagnosis was significantly associated with inferior overall and cancer-specific survival outcomes, underscoring the significance of timely surgical management in this setting.

Given that the ongoing COVID-19 pandemic may limit hospital resources, these findings emphasize the importance of prioritizing prompt definitive treatment for patients with urothelial cancers. Such interventions should be performed expeditiously to achieve the best possible oncologic outcomes in patients with these malignancies, which are known to have a propensity for aggressive behavior and progression [41]. Therefore, it is imperative to avoid undue delays in surgical intervention and expedite treatment when possible, even during times of limited hospital resources [42].

In our study, it was observed that the proportion of MIBC cases increased significantly during the COVID-19 pandemic. Trimodal therapy and RC are the two primary treatment options for patients with MIBC [43]. Trimodal therapy includes a combination of transurethral resection of the bladder tumor, chemotherapy, and radiotherapy, while RC involves the surgical removal of the bladder. In the pandemic context, there are several factors to consider when deciding between these two treatment options. One key factor is patient choice, as some patients may prefer one treatment option over the other due to concerns about exposure to the virus in healthcare settings or the risk of complications associated with surgery. Additionally, access to radiotherapy services may be limited during the pandemic, which could affect the feasibility of trimodal therapy for some patients. Several studies have compared the effectiveness of trimodal therapy and RC for MIBC, and the results are mixed. Some studies have shown that trimodal therapy can provide similar survival outcomes to RC for select patients with MIBC [44]. However, other studies have suggested that RC may provide better long-term outcomes, particularly for patients with high-risk disease [45]. Overall, the decision between trimodal therapy and RC should be based on individual patient factors, including disease stage, patient preferences, and the availability of healthcare resources. Additional research is needed to determine the optimal management approach for MIBC patients during the pandemic and beyond.

A worrying outcome of this investigation is the increased incidence of invasive cancer in both newly diagnosed and recurrent patients during the pandemic period. This differs from the statistics provided by a study of more than 700 individuals who had surgical intervention for urothelial cancer in 2019 and 2020 and found no variations in cancer stage or severity between the two years [46]. Nevertheless, it is crucial to highlight that at baseline, 50% of patients in this study had an invasive form of cancer, and 72% had high-grade cancer, compared to 50% and 72%, respectively, in our analysis. In addition, while no variations in disease stage or severity were seen among those receiving TURBT, a statistically significant increase in node-positive and non-organ-confined disease was observed in patients who underwent RC after the pandemic, compared with the previous year. This also confirms our results that individuals receiving chemotherapy for urothelial cancer had worse histological characteristics after the pandemic.

COVID-19 had a major influence on elective surgical activity at our facility, as it did globally [47]. Multiple policies have been created at various institutions to guarantee that non-COVID patients continue to have access to emergency treatment. Critical oncology cases were transferred to nearby hospitals in the peak months of COVID-19 when the percentage of COVID-positive patients stayed high. As infection numbers started to decline, our facility resumed elective surgery for urgent and time-sensitive situations. In our facility, all urothelial cancer patients awaiting surgery were prioritized as emergency and time-sensitive. Consequently, the waiting times for treatment remained relatively the same between the two intervals of time examined in this research. As the period between symptom start and admission was unknown, waiting durations were computed as the time between presentation and treatment using the existing evidence.

The COVID-19 pandemic should not impede the diagnostic examination of symptoms associated with bladder cancer. In addition, RC should be scheduled within 10–12 weeks after diagnosis and neoadjuvant chemotherapy [48]. When feasible, neoadjuvant chemotherapy should be explored since it provides a survival advantage while waiting for the optimal moment to undertake an oncologic excision treatment. There is also sufficient evidence that urothelial cancer permits a longer deferral time than bladder urothelial cancer, which may be crucial in the event of a worldwide lockdown or suspension of elective surgical procedures. Patients with urothelial cancer should be recruited in immunization programs globally in order to protect them adequately against COVID-19 infection, morbidity, and death. This technique also enables patients to keep their planned appointments for endoscopic follow-up and physician visits, as well as to continue with their treatment plans.

### 4.2. Study Limitations and Future Perspectives

This research examines the differences in demographic factors, clinical, and surgical outcomes of patients with urothelial cancer in Romania. Because of the potential for human error in the creation of a digital database from patients’ paper records, the quality of the data that was evaluated in a retrospective cohort approach may have been lower than expected. Another limitation of the current study is that it had a limited sample size since it was collected only from one hospital. As a consequence, it is possible that the features and outcomes of those who were diagnosed with urothelial cancer in Romania during the COVID-19 pandemic are not fully and appropriately reflected by these data. Even though it was plausible to assess only the progression of urothelial cancer at six months, another constraint is the fairly short follow-up time frame that did not provide for a proper assessment of the pandemic’s consequences on disease-free survival and total survival rates. This is despite the fact that it was possible to compare only the progression of the disease. The possibility of SARS-CoV-2 infection and the increase in COVID-19 patients may have had a negative effect on the capability of the registry or the quality of the collected data.

Nevertheless, this study offers strong evidence that the COVID-19 pandemic in Romania was not a substantial risk factor for short-term survival and disease progression among patients diagnosed with urothelial cancer. As a result, the fundamental consideration of information presented in this study is the consolidation of existing data, as well as endorsing guidelines that limit the period of treatment delay. This timeframe of treatment postponement should not compromise potential outcomes and survival in urothelial cancer patients who are treated in surgical departments that have been reorganized to support COVID-19 patients. As a result, research must be conducted from several centers with a high number of participants in order to discover the complete range of effects brought about by the ongoing pandemic.

## 5. Conclusions

The year 2020 was characterized by major changes in the healthcare sector due to adaptations imposed by the COVID-19 pandemic, determining a significant decrease in the number of elective interventions in the urology department and more patients with urothelial carcinoma presenting in later stages or in emergent presentations such as bladder obstruction or hemorrhagic cystitis. The decrease in patient presentations for diagnosis and treatment of urothelial cancer probably determined the significant rise in patients in the following year of 2021. However, the number of patients with advanced urothelial cancer and muscle-invasive bladder cancer was significantly higher than the year prior to the COVID-19 pandemic. Therefore, we advocate for increased public health awareness of urothelial cancer and increased attention toward the screening and management of these patients in the following years.

## Figures and Tables

**Table 1:** Background of patients with urothelial carcinoma sorted by the year of diagnosis and intervention.

	2019 (*n* = 421)	2020 (*n* = 326)	2021 (*n* = 375)	*p*-Value *
Background				
Age, years (mean ± SD) **	63.2 ± 9.6	64.0 ± 10.1	64.4 ± 10.3	0.224
Age range				0.356
<50	88 (20.9%)	81 (24.8%)	76 (20.3%)	
50–70	175 (41.6%)	143 (43.9%)	164 (43.7%)	
≥71	158 (37.5%)	102 (31.3%)	135 (36.0%)	
Gender				0.911
Female	186 (44.2%)	142 (43.6%)	160 (42.7%)	
Male	235 (55.8%)	184 (56.4%)	215 (57.3%)	
BMI, kg/m^2^ (mean ± SD)	26.6 ± 3.7	27.2 ± 3.9	27.0 ± 4.1	0.097
Substance use behavior				
Chronic smoking	149 (35.4%)	132 (40.5%)	141 (37.6%)	0.361
Chronic alcohol use	32 (7.6%)	28 (8.6%)	25 (6.7%)	0.630
Place of origin				0.436
Rural	182 (43.2%)	155 (47.5%)	163 (43.5%)	
Urban	239 (56.8%)	171 (52.5%)	212 (56.5%)	
Referred from				0.090
Primary care	194 (46.1%)	127 (39.0%)	173 (46.1%)	
Secondary care	227 (53.9%)	199 (61.0%)	202 (53.9%)	
SARS-CoV-2 infection	-	17 (5.2%)	13 (3.5%)	0.254
COVID-19 vaccination status				<0.001
Yes	-	37 (11.3%)	94 (25.1%)	
No	-	289 (88.7%)	281 (74.9%)	

* Chi-square or Fisher’s exact test; ** ANOVA test; SD Standard Deviation; BMI—Body Mass Index.

**Table 2:** Comparison of urothelial cancer characteristics during the study period.

	2019 (*n* = 421)	2020 (*n* = 326)	2021 (*n* = 375)	*p*-Value
Number of comorbidities				0.150
0-1	116 (27.6%)	103 (31.6%)	98 (26.1%)	
2	162 (38.5%)	119 (36.5%)	127 (33.9%)	
≥3	143 (34.0%)	104 (31.9%)	150 (40.0%)	
Anatomical distribution				0.939
Renal pelvis and ureter	83 (19.7%)	61 (18.7%)	73 (19.5%)	
Bladder	338 (80.3%)	265 (81.3%)	302 (80.5%)	
Tumoral infiltration	(*n* = 338)	(*n* = 265)	(*n* = 302)	0.046
NMIBC	235 (69.5%)	166 (62.6%)	183 (60.6%)	
MIBC	103 (30.5%)	99 (37.4%)	119 (39.4%)	
Grading				0.081
Low grade	149 (35.4%)	127 (39.0%)	131 (34.9%)	
High grade	144 (34.2%)	116 (35.6%)	112 (29.9%)	
Unknown	128 (30.4%)	83 (25.5%)	132 (35.2%)	
TNM staging				<0.001
Stage 0a-0is	104 (24.7%)	59 (18.1%)	66 (17.6%)	
Stage I	175 (39.2%)	129 (39.6%)	169 (45.1%)	
Stage II	85 (20.2%)	76 (23.3%)	54 (14.4%)	
Stage III	46 (10.9%)	54 (16.6%)	57 (15.2%)	
Stage IV	11 (2.6%)	8 (2.5%)	29 (7.7%)	
Invasion				<0.001
Local invasion	131 (31.1%)	130 (39.9%)	140 (37.3%)	
Distant invasion	11 (2.6%)	8 (2.5%)	29 (7.7%)	

Data were analyzed using Chi-square or Fisher’s exact test; TNM—Tumor Node Metastasis; NMBIC—Non-Muscle-Invasive Bladder Cancer; MIBC—Muscle-Invasive Bladder Cancer.

**Table 3:** Comparison of urothelial cancer management during the study period.

	2019 (*n* = 421)	2020 (*n* = 326)	2021 (*n* = 375)	*p*-Value *
Intervention				
Cystectomy	22 (5.2%)	14 (4.3%)	38 (10.1%)	0.003
TURBT	368 (87.4%)	289 (88.7%)	292 (77.9%)	<0.001
Nephrectomy	31 (7.4%)	23 (7.1%)	45 (12.0%)	0.028
Neoadjuvant chemotherapy	86 (20.4%)	71 (21.8%)	89 (23.7%)	0.529
Radiotherapy	48 (11.4%)	36 (11.0%)	54 (14.4%)	0.312
Emergency presentation	(*n* = 59)	(*n* = 67)	(*n* = 73)	0.038
Urinary obstruction	48 (11.4%)	52 (16.0%)	55 (14.7%)	
Hemorrhagic cystitis	11 (2.6%)	15 (4.6%)	18 (4.8%)	
Outcomes				
Days of hospitalization	9.8 ± 3.9	8.3 ± 4.1	8.5 ± 4.0	<0.001
In-hospital mortality	13 (3.1%)	16 (4.9%)	14 (3.7%)	0.434
Disease progression at six months	37 (8.8%)	35 (10.7%)	44 (11.7%)	0.380

* Chi-square or Fisher’s exact test; TURBT—Trans Urethral Resection of Bladder Tumor.

**Table 4:** Risk factors for urothelial cancer progression after the initial hospital visit.

Risk Factors	HR	CI	*p*-Value
Advanced TNM stage (III + IV)	5.61	2.29–6.90	<0.001
Distant invasion	5.13	3.06–7.08	<0.001
MIBC type	2.49	1.52–4.26	0.001
Emergency hospitalization	1.32	1.04–3.17	0.009
Duration of hospitalization	1.27	0.95–2.03	0.166
Year of admission (2020)	1.13	0.90–1.76	0.183
Year of admission (2021)	1.04	0.87–1.92	0.242

TNM—Tumor Node Metastasis cancer staging system; MIBC—Muscle-invasive bladder cancer; HR—Hazard Ratio; CI—Confidence Interval.

## Data Availability

The data presented in this study are available on request from the corresponding author.
